# Which individual, social, and urban factors in early childhood predict psychopathology in later childhood, adolescence and young adulthood? A systematic review

**DOI:** 10.1016/j.ssmph.2023.101575

**Published:** 2023-12-09

**Authors:** Daniele Franzoi, Claudi L. Bockting, Kirsty F. Bennett, Annick Odom, Paul J. Lucassen, Alisha Pathania, Alexandra Lee, Marlies E. Brouwer, Rens van de Schoot, Reinout W. Wiers, Josefien J.F. Breedvelt

**Affiliations:** a Department of Psychiatry, Amsterdam Public Health (APH), Amsterdam University Medical Centres, University of Amsterdam, the Netherlands; b Centre for Urban Mental Health, University of Amsterdam, Amsterdam, the Netherlands; c The National Centre for Social Research, London, UK; d Faculty of Social and Behavioural Sciences, University of Amsterdam, the Netherlands; e Department of Methodology and Statistics, Faculty of Social and Behavioral Sciences, Utrecht University, the Netherlands; f Brain Plasticity Group, Swammerdam Institute for Life Sciences, University of Amsterdam, the Netherlands; g Department of Child and Adolescent Psychiatry, Institute of Psychiatry, Psychology and Neuroscience, King's College London, UK

## Abstract

**Background:**

A comprehensive picture is lacking of the impact of early childhood (age 0–5) risk factors on the subsequent development of mental health symptoms.

**Objective:**

In this systematic review, we investigated which individual, social and urban factors, experienced in early childhood, contribute to the development of later anxiety and depression, behavioural problems, and internalising and externalising symptoms in youth.

**Methods:**

Embase, MEDLINE, Scopus, and PsycInfo were searched on the 5^th^ of January 2022. Three additional databases were retrieved from a mega-systematic review source that focused on the identification of both risk and protective indicators for the onset and maintenance of prospective depressive, anxiety and substance use disorders. A total of 46,450 records were identified and screened in ASReview, an AI-aided systematic review tool. We included studies with experimental, quasi-experimental, prospective and longitudinal study designs, while studies that focused on biological and genetical factors, were excluded.

**Results:**

Twenty studies were included. The majority of studies explored individual-level risk factors (N = 16). Eleven studies also explored social risk factors and three studied urban risk factors. We found evidence for early predictors relating to later psychopathology measures (i.e., anxiety and depression, behavioural problems, and internalising and externalising symptoms) in childhood, adolescence and early adulthood. These were: parental psychopathology, exposure to parental physical and verbal violence and social and neighbourhood disadvantage.

**Conclusions:**

Very young children are exposed to a complex mix of risk factors, which operate at different levels and influence children at different time points. The urban environment appears to have an effect on psychopathology but it is understudied compared to individual-level factors. Moreover, we need more research exploring the interaction between individual, social and urban factors.

## 1 Introduction

There is a vast increase of people expected to live in cities in the future (The World Bank, 2018). By 2050, approximately 70% of the world's population is expected to live in an urban area (The World Bank, 2018).

The effects of increasing urbanisation on mental health are yet to be fully disentangled. Incidence rates of mood disorders have been reported to be higher among people living in urban areas compared to people living in rural areas (Vassos et al., 2016; Wiens et al., 2017). It appears that urban living increases the risk of depression and anxiety, however, full causal effects are still to be established (Evans et al., 2018; Okkels et al., 2018; Van der Wal et al., 2021). The future anticipated rise in urbanisation will most likely affect our younger generations, which underscores the importance of disentangling the potential effect of urbanisation on children and young people.

Very young children, including infants (aged 0 to 5) in particular, may be susceptible to adverse environmental exposures commonly associated with urban living (Doom & Gunnar, 2013). For instance, exposure at this stage to food poverty, a lack of parental attachment and/or maternal stress can have notable effects on the development of mental health conditions (e.g. depression and anxiety) later in life (Balbernie, 2013; Conroy & Brown, 2004; Reemst et al., 2022).

In order to identify the unique contribution of urban factors, Van der Wal et al. (2021) introduced a conceptual framework of individual (e.g. gender, age, cognitive and neurobiological factors), social (e.g. neighbourhood social cohesion) and urban factors (ambient or physical urban environment). To date, research suggests that social factors, such as a lack of social cohesion, crime victimisation, trust in the community and low socio-economic status, as well as urban stressors (e.g., noise, pollution) are important risk factors for depression and anxiety (Breedvelt et al., 2022; Caspi et al., 2000; Galea et al., 2011; Manca, 2014; Van der Wal 2021).

Specific urban and social factors can also be protective, for instance the presence of green spaces was shown to correlate with an improved social cohesion (i.e. a social factor) and reduce pollution and urban heat (as urban factors), which in turn, have been reported to improve mental health in adults (Coventry et al., 2021; South et al., 2018; Van den Berg et al., 2016; Vanaken & Danckaerts, 2018). In addition, by actively reducing noise and air pollution (as urban factor) and by facilitating social interactions and options for exercise (i.e. social and individual factors), psychological resilience may be further increased from adolescence into adulthood (Engemann et al., 2019).

As highlighted above, the urban, social and individual factors are intrinsically linked, and very young children are not solely exposed to urban stressors nested within the built environment, such as noise and air pollution, but also to social stressors, like poverty and lack of social support or social capital, that can modulate their mental health development, and ultimately interact with their upbringing in an urban environment (Krabbendam et al., 2021). In this review, we aim to provide a comprehensive overview of the relative effects of individual, social and urban factors during early childhood, on later psychopathology.

Indeed, while prior studies and reviews have indicated a range of psychological and social risk factors contributing to the development of mental health conditions in late adolescence and young adulthood, the focus has so far primarily been on risk factors at the individual-level (e.g. parental mental health) but has not yet focused on very young children (Goodman et al., 2011; Herba et al., 2016). Moreover, while social factors and environmental factors have been explored (such as adverse childhood experiences (Scully et al., 2020; Walker et al., 2011), socioeconomic disadvantage (Reiss, 2013) and racism (Berry et al., 2021)), we lack a comprehensive overview of the relative effects of all early childhood factors on later psychopathology in young children.

In addition, the aforementioned factors and reviews omit the potential interactions of factors across different (time) scales. Taking a complex systems perspective of mental health science, we set out to explore the range of factors at the urban level (i.e. urban institutions and indicators), the social level (community-level institutions) and the individual level (e.g., individual demographics or the family environment, and how they interact with each other over time (Van der Wal et al., 2021).

This systematic review explores the evidence for individual, social and urban factors in early childhood (age 0–5) on the development of psychopathology (i.e. anxiety and depression, internalising and externalising symptoms, behavioural problems, and wellbeing) in later childhood (age 5–11), adolescence (age 12–20) and young adulthood (age 20–25).

## 2 Methods

This review was registered with PROSPERO (CRD42022302280) and followed the Preferred Reporting Items for Systematic Review and Meta-Analysis (PRISMA) guidelines (Page et al., 2021).

### 2.1 Search strategy

We utilised an adapted search strategy from a recent systematic review studying predictors of depression, anxiety and substance use disorder across the lifecourse (the ‘ASmental review Urban mental Health project’; Brouwer et al., 2022). The search terms were linked using Boolean operators (see Appendix 1 for search strategy). The search was conducted in PsycInfo, Embase, Scopus and MEDLINE on the 5^th^ of January 2022.

### 2.2 Inclusion criteria

Longitudinal study designs were included. Cross-sectional, retrospective, case-control, review, qualitative or other, non-prospective or non-longitudinal studies were excluded. As the focus of this review was amenable risk factors, studies that focused on predictors related to biology and genetics were excluded (e.g. genetic vulnerability, birth weight and pre-term birth). This choice was made to test the association between the environment, in its broader conception which spans across individual, social, and urban levels. Studies were included if the exposure factor(s), defined as a factor measured during early childhood and included in the analysis, was present and measured between 0 and 5 years of age. As criteria for inclusion, the mean age of exposure to the factor(s) and outcome(s) measured were used. Studies were included if they used a validated questionnaire (e.g. Strengths and Difficulties Questionnaire (SDQ) and the Warwick-Edinburgh Mental Wellbeing Scale (WEMWBS)) or diagnostic interview to assess one of the following outcomes up to 25 years of age: psychopathology, (i.e. anxiety, depression, internalising symptoms and externalising symptoms, and behavioural difficulties) and wellbeing.

### 2.3 Screening for eligible studies

After de-duplication in EndNote, a machine learning-aided software, ASReview Version 0.19 (ASReview LAB developers, 2022), which applies active learning to the process of screening articles, was used to screen the search results (van de Schoot et al., 2021). Three additional databases from the ASmental review project were also uploaded. These three databases contained articles assessing prospective risk factors for depression, anxiety and substance use disorders. To reduce risk of rank-order bias (Norman et al., 2020), ASReview utilised three separate screening phases (active learning, neural net and review of excluded papers). Such a multi-phase screening process goes beyond screening process that goes beyond the standard PRISMA criteria for AI-aided screening with prioritization (Page et al., 2021). The default model was selected in ASReview, and a stopping rule of 5% was chosen for the search database, whereas a stopping rule of 10–11% was chosen for the three mega-meta databases due to their smaller number of articles. Random prior knowledge was used to initially train ASReview for the mega-meta databases, whereas for the search databases, 5 articles that met our inclusion criteria were manually searched as prior knowledge. Two researchers (AO and DF) independently screened the titles and abstracts of articles. Relevant full texts were then screened by DF and AO. Any discrepancies were discussed with a third researcher (KB). The final list of included studies was checked for appropriateness of inclusion by KB and JB.

### 2.4 Data extraction

Three researchers (AO, AP, and DF) independently extracted data from articles. Data extracted included age, exposure factor, outcome measurement and results. Another researcher not involved in the data extraction (KB) checked 10% of the extracted articles. Information about internalising/externalising disorders, behavioural problems, psychopathology, and diagnosis of depression and anxiety were extracted. Information on exposure indicators (individual, social, and urban factors) was also extracted. Examples of factors extracted include parental psychopathology, family socioeconomic status and neighbourhood deprivation.

### 2.5 Quality assessment

The quality of included studies was assessed using the Critical Appraisal Skills Programme checklist for cohort studies (CASP, 2022) by three independent researchers (AO, AP, DF)*.* CASP is a 12-item checklist assessing the quality and validity of a study. CASP items assess risk of measurement bias and accuracy of results, as well as adequacy of sample size and follow-up time. Studies were considered high quality if no risk of measurement bias was present, results were reported correctly (i.e. coefficients and confidence intervals provided), and the sample was recruited systematically. If any of these elements were rated as “no”, the study was considered of medium quality, and if more than one element was absent the study was rated as low quality.

### 2.6 Data synthesis

A narrative synthesis of results was carried out where studies were grouped by factors based on the framework provided by Van der Wal et al. (2021): individual, social, and urban factors. This framework was chosen as it provides categories which divide micro, meso and macro risk factors for mental health difficulties. Within each indicator category, outcomes were subdivided according to age at time measurement. Four subcategories were created: childhood (0–5), middle childhood (age 6–11), adolescence (age 12–19) and early adulthood (age 20–25).

## 3 Results

### 3.1 Search results

**Fig. 1:**
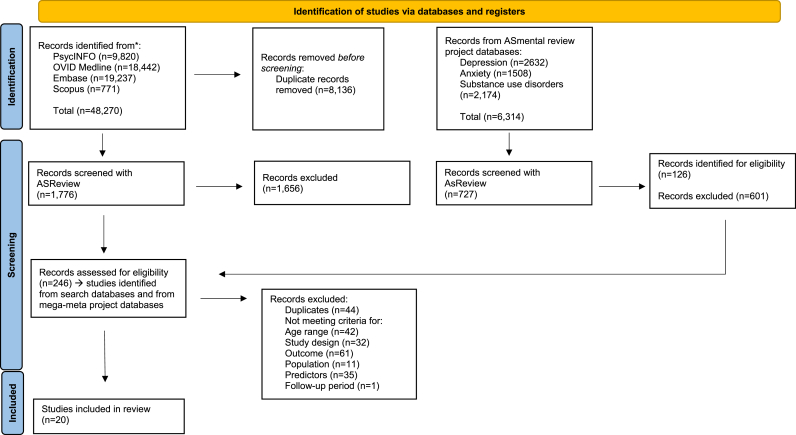
PRISMA flowchart of studies selection.

We retrieved 40,136 citations from the search databases and an additional 6314 from the previously described mega-meta review (Brouwer et al., 2022) (anxiety n = 1508; substance use n = 2174; depression n = 2632), resulting in a total of 46,450 articles. Among the four databases, a total of 2503 titles and abstracts (7.96%) were screened with ASReview (database search n = 1776; anxiety n = 200; depression n = 264; substance use n = 263), and of these, 246 studies met the inclusion criteria. The included batch of articles underwent another round of full-text screening, after which 226 articles were excluded, leading to a final inclusion of 20 articles. Fig. 1 shows the PRISMA-P flowchart.

### 3.2 Characteristics of included studies

Studies were conducted in the USA and Canada (n = 10), Europe (n = 8) and Australia and New Zealand (n = 6). Of these studies, 17 assessed outcomes with a validated questionnaire and 3 used a diagnostic interview. Outcomes were measured in participants between 2 and 21 years of age, and follow-up measurements were made between 1 and 21 years of age (see Table 1, baseline characteristics of included studies). Outcomes included anxiety (n = 9), depression (n = 6), behavioural problems (n = 6), internalising problems (n = 6), externalising problems (n = 4), conduct problems (n = 2), psychiatric morbidity (n = 1) and children's mental health (n = 1). Some studies assessed more than one outcome (n = 10).

**Table 1:** Baseline characteristics of included longitudinal studies.

Study	Study location	Sample recruitment	Sample description	Percentage female in included sample	Ethnicity
Bakoula et al. (2009)	Greece	Recruitment from The Greek Birth Cohort, based on the Greek National Perinatal Survey, a prospective study of all the 11,048 births throughout Greece between the 1^st^and the 30th of April 1983.	n = 2065 children	56.27%	Not reported (N/R)
Barrios et al. (2018)	USA	Recruitment from a longitudinal study examining young children's neuroendocrine function and risk for depression	n = 108 pre-school age children	50%	54% White; 31% Black/African American; 13% Multiracial; 10% Other.
Bjorkenstam et al., 2014	USA	Oversample of African American families and with low income	n = 2223 children born between 1985 and 1995	N/R	67% white non-Hispanic; 17% black non-Hispanic; 13% Hispanic
Carter et al. (2010)	USA	Recruitment from birth records New Haven–Meriden Standard Metropolitan Statistical Area of the 1990 Census.	n = 442 elementary school children with social-emotional/behavioural problems	50.50%	74.4% Caucasian; 23.7% African American; Hispanic 1.9%
Christensen et al. (2017)	Australia	Recruitment from the Longitudinal Study of Australian Children (LSAC)	n = 3371 children representative of the general Australian population	N/R	N/R
Cohen and Bromet (1992)	USA	Mothers living near Three Mile Island, Pennsylvania.	n = 620 children with onset of behavioural problems, n = 71 children with recurrence of behavioural problems	N/R	N/R
Comeau and Boyle (2018)	Canada	Sample was drawn from the National Longitudinal Survey of Youth	n = 1553 children always poor; n = 2355 children intermittently poor.	N/R	*Always poor*: 53.6% African American; 28.1% Hispanic; 18.3% non-African American and non- Hispanic white. *Intermittently poor*: 24.7% African American; 20% Hispanic; 55.3% non-African American and non- Hispanic white.
D'Souza et al. (2019)	New Zealand	Recruitment from the *Growing Up in New Zealand* birth cohort who were born between 25 April 2009 and 25 March 2010.	n = 5885 children	N/R	N/R
Dunn et al. (2020)	The Netherlands	Data come from Generation R, a population-based prospective study based in Rotterdam, the Netherlands.	n = 9778 children followed from foetal life onward	50.70%	65.5% Dutch; 7.75% non-Dutch European; 25.72% Non-Dutch non-European
Fitzsimons et al. (2017)	UK	Recruitment from the Millennium cohort study, born between 2000 and 2001	n = 11049 children	N/R	N/R
Hayatbakhsh et al. (2012)	Australia	Recruitment from the 21-year Mater-University of Queensland Study of Pregnancy (MUSP), a study conducted at the Mater Misericordiae Hospital in Brisbane, Australia, between 1981 and 1983.	n = 4502 women who gave birth to a live singleton baby	48.50%	N/R
Hyland et al. (2016)	Denmark	Recruitment through Danish Civil Registration System (CRS) and the Danish Psychiatric Central Register	n = 54458 Danish children	51.10%	N/R
Karevold et al. (2009)	Norway	Families attending child health clinic for vaccination in 19 health care areas of Norway, thought to represent the diversity of the Norwegian social environments	n = 941 Norwegian families	54%	Sample was predominantly ethnic Norwegian families (representative of ethnic distribution in Norway, as 2.3% of the population come from non-western countries)
Kiernan & Mensah, 2009	UK	Recruitment from the Millennium cohort study, born between 2000 and 2001	n = 14777 families from two waves of the Millennium Cohort Study	49.2%	N/R – only reported that “allowed for over-representation of families living in areas of England with high rates of child poverty or high proportions of ethnic minorities, and the three smaller countries of the UK"
King et al., 2018	USA	Recruitment from the Fragile Families and Child Wellbeing Study (FFCWS) who were born between 1998 and 2000 in 20 large U.S. cities	n = 2044 children	N/R	Food secure household: 23.9% white; 51.1% Black; 21.7% Hispanic; 3.3% other. Food insecure household: 16.2% white; 59.3% Black; 21.7% Hispanic; 3.3% other. Food secure child: 22.8% white; 52.3% Black; 21.7% Hispanic; 3.2% other. Food insecure child: 19% White; 57.1% Black; 20.2% Hispanic; 3.7% Other.
Lai et al. (2019)	UK	Recruitment from the Millennium cohort study, born between 2000 and 2001	n = 10329 total sample size of children from the Millennium cohort study. n = 6652 were never in poverty, whereas n = 1424 were in poverty in early childhood. n = 2576 children not included in this review as poverty was measured after 5 years of age.	53%	*Never in poverty*: 92.8% white; 0.5% mixed; 2.4% Indian; 0.9% Pakistani and Bangladeshi; 1.5% Black; 1.5% other. *Poverty in early childhood*: 84.1% white; 1.3% mixed; 4% Indian; 3.5% Pakistani and Bangladeshi; 4.5% Black; 2.6% other
Leech et al. (2006)	USA	Women recruited sequentially from a prenatal clinic	n = 829 women 18 years old and older who were in their fourth or fifth prenatal month	50%	47% White; 53% Other.
Martikainen et al. (2018)	Finland	Parents and offspring data come from hospital discharge records maintained by the National Institute for Health and Welfare for 1986–2011	Individuals born in years 1986–1996 (n = 136,604) and followed from the beginning of the year of their 15th birthday until first incidence of psychiatric morbidity, the end of the year of their 25th birthday, emigration, death, or the end of year 2011	N/R	N/R
Najman et al. (2010)	Australia	Recruitment from Mater Hospital– University of Queensland Study of Pregnancy (MUSP), a prospective longitudinal study of a consecutive cohort of individuals born in Brisbane, Australia, between 1981 and 1984 at a major public hospital	n = 3588 children	N/R	N/R
Shaw et al. (2016)	USA	Families recruited from the Allegheny County WIC program in the Pittsburgh Metropolitan area when boys were 1.5 years of age	Study 1 is an all-male, ethnically diverse, low-income sample of n = 310 boys and mothers from a large urban context; Study 2 provides a larger cohort of n = 731 ethnically diverse, low-income boys and girls from urban, rural, and suburban communities.	Study 1: 0%; Study 2: 49%	Study 1: 53% European American; 36% African American; 5% biracial; 6% other. Study 2: 50% European American; 28% African American; 13% biracial; 9% other.

*N/R = Not Reported.

### 3.3 Risk of bias

The majority of the studies assessing individual-level risk factors, such as parental psychopathology, family demographics and violence exposure were rated as high quality (n = 16/18). Two studies rated as low quality had a risk of measurement bias of exposure indicators and outcomes. Moreover, confidence in the results reported was reduced as confidence intervals were not reported for these two studies (Cohen & Bromet, 1992; Kiernan & Mensah, 2009). Studies assessing social-level risk factors were nearly all rated as high quality (n = 9/11). Two studies were rated as medium quality as the risk factors measured, total annual gross income and maternal depression, were assessed with a self-report questionnaire (Kiernan & Mensah, 2009; Najman et al., 2010). Self-reported annual income might have introduced bias as the income was not verified, and maternal depression would have been more reliable if measured with a validated questionnaire. All three studies assessing urban-level risk factors were rated as high quality as no risk of measurement bias was present, results were reported precisely and the sample was recruited systematically. Most of the findings in the review were rated as high quality (n = 17/20). The sample sizes of the included studies were large enough to be able to generalise the findings and follow-up times were long enough to assess longitudinal associations.

### 3.4 Results by individual, social and urban factors

Table 2 provides a summary overview of the individual, social and urban factors and their association with mental health outcomes. Table 3 provides a more detailed overview of individual study results.

**Table 2:** Summary of associations between main exposure risk factors and onset of psychopathology in children (age 5–11), adolescents (age 12–19), and young adults (age 20–25).

Main risk factors	Level (individual, social or urban)	Outcome	Studies
Parental psychopathology	Individual	Increased risk of developing depression and anxiety in childhood, adolescence, and young adulthood	Hyland et al., 2016; Karevold et al., 2009; Leech et al., 2006; Hayatbakhsh et al., 2012
Parental psychopathology	Individual	Increased risk of behavioral problems in childhood and adolescence	Kiernan & Mensah, 2009, Cohen & Bromet, 1992; Christensen et al., 2017; Hayatbakhsh et al., 2012
Poverty	Social	Increased risk of behavioral problems in adolescence	Christensen et al., 2017; Lai et al., 2019
Poverty	Social	Increased risk of developing anxiety and depression in young adulthood	Björkenstam et al., 2017; Najman et al., 2010
Urbanicity	Urban	Increased risk of anxiety in adolescence and young adulthood	Hyland et al. (2016)

**Table 3:** Individual, social, and urban risk factors for mental health developmental outcomes.

Study	Predictor	Level (Invidual, Social, Urban)	Predictor sub-type	Measurement of predictor	Outcome	Measurement of outcome	Results
Bakoula et al., 2009	Predictor: Birth Outcome: 7 years	Individual	Type of birth delivery	Birth records	Children's mental health (onset)	Rutter's Parent Questionnaire A2	Assisted delivery was associated with conduct problems (t = −1.98, p < .05), but not with emotional problems (t = 0.61, p > .05). Caesarean section was not associated with emotional (t = −1.01, p > .05) or conduct (t = 0.04, p > .05) problems.
			Maternal distress at birth				Maternal distress during delivery was not associated with emotional (t = −0.55, p > .05) or conduct (t = 0.57, p > .05) problems.
			Child peripartum distress				Child peripartum distress was not associated with emotional (t = −0.21, p > .05) or conduct (t = −1.60, p > .05) problems.
Barrios et al. (2018)	Predictor: 3–5 years Outcome: 6–8 years	Individual	Reinforcing parent-child interaction	Parent-Child Sleep Interaction Scale (PSIS)	Depression/Anxiety (onset)	PAPA (Preschool Age Psychiatric Assessment)	Children with more sleep problems and higher PSIS Sleep Reinforcement scores at T1 showed increases in depressive (p = .057) and anxiety (p = .010) symptoms at T2.
			Conflict parent-child interaction				PSIS Sleep Conflict at T1 did not significantly predict increases in children's depressive and anxiety symptoms at T2 (p = ns).
			Dependence parent-child interaction				PSIS Sleep Dependence at T1 did not significantly predict increases in children's depressive and anxiety symptoms at T2 (p = ns).
			Parent-child sleep interactions				PSIS Total at T1 did not significantly predict increases in children's depressive and anxiety symptoms at T2 (p = ns).
Bjorkenstam et al., 2017	Predictor: 0–6.9 years Outcome: 12–17 years	Individual/Social	Household receiving public assistance	Including food stamps, Aid to Families with Dependent Children (AFDC)/Temporary Assessment for Needy Families (TANF), supplementary security income	Conduct/internalising problems, depression (onset)	Child Behavioural Checklist (CBCL)/Internalising Index/Children's Depression Inventory (CDI)	Household public assistance was associated with the incidence of adolescent depressive symptoms (IRR = 1.1, 95% CI = 0.9–1.4).
			Long-term parental unemployment	Unemployment defined as a spell of at least six months in the past year			Long-term parental unemployment was associated with the incidence of adolescent depressive symptoms (IRR = 0.7, 95% CI = 0.5–1.2).
			Residential instability	Three or more family moves			Residential instability was associated with the incidence of adolescent depressive symptoms (IRR = 1.3, 95% CI = 1.0–1.7).
			Poverty	Total household income divided by the official US poverty threshold corresponding to the size of the given household			Poverty was associated with the incidence of adolescent depressive symptoms (IRR = 0.9, 95% CI = 0.6–1.1).
			Single parent Household	Asked whether parent lived with a partner			Single parent household was associated with the incidence of adolescent depressive symptoms (IRR = 1.3, 95% CI = 1.0–1.7).
Carter et al. (2010)	Predictor: 12–36 months Outcome: 3–9 years	Individual/Social	Child's stressful life events	Life events Inventory	Externalising/internalising disorders (onset)	Diagnostic Interview Schedule for Children, Version IV	The likelihood of externalising disorders was greater among children who had experienced stressful life events (OR = 3.76, 95% CI = 1.27–11.16, p = .0115)
			Low family expressiveness	Expressiveness and Conflict subscales of the Family Environment Scale			The likelihood of externalising disorders was greater among children who had low family expressiveness (OR = 3.19, 95% CI = 1.11–9.21, p = .0245).
			Parental low education	Vineland Screener semi-structured interview			Children with internalising disorders were more likely than children with no disorder (OR = 3.7, 95% CI = 1.34–10.25, p = .0079) or externalising disorders (OR = 5.6, 95% CI = 1.38–22.50, p = .0077) to have parents with lower education.
			Child's violence exposure	Child Life Events and Violence Exposure scales			Children with internalising disorders were more likely than those with no disorder (OR = 8.93, 95% CI = 3.03–26.31, p < .0001) and externalising disorders (OR = 5.45, 95% CI = 1.45–20.46, p = .0047) to have been exposed to violence.
			Not being minority race/ethnicity	Birth records			The likelihood of externalising disorders was greater among children who were not of minority race/ethnicity (OR = 2.20, 95% CI = 1.01 to 4.78, p = .0373).
Christensen et al. (2017)	Predictor: 4–5 years Outcome: 14–15 years	Individual/Social/Urban	Warmth/Hostility/Persistence - maternal parenting	Self-report measures developed for Longitudinal Study of Australian Children (LSAC)	Childhood behavioural difficulties (onset)	Strengths and difficulties questionnaire (SDQ)	Maternal warmth (OR = -0.43, p < .05), maternal hostility (OR = 0.82, p < .001), and maternal persistence (OR = 2.05, p < .001) were associated with behavioural difficulties at age 14.
			Maternal psychological distress	Kessler 6 (K6)			Maternal psychological distress was associated with behavioural difficulties at age 14 (OR = 1.87, p < .001).
			Maternal education	Self-report question			Maternal education was associated with behavioural difficulties at age 14 (OR = 1.06, p < .001).
			Weekly family income				Weekly family income was associated with behavioural difficulties at age 14 (OR = 1.22, p < .001).
			Economic and social disadvantage in small areas	Neighbourhood socio-economic index for areas (SEIFA)			Economic and social disadvantage was associated with behavioural difficulties at age 14 (OR = 0.89, p < .001).
Cohen and Bromet (1992)	Predictor: 3 years Outcome: 4 years	Individual/Social	Maternal age	Diagnostic interview N/R	Child behavioural problems (onset and recurrence)	Conners' Parent rating scale	Maternal age was not associated with the onset (r = −.05, p > .05) or recurrence (r = .06, p > .05) of behavioural problems.
			Maternal education				Maternal education was not associated with the onset (r = −.07, p > .05) or recurrence (r = −.01, p > .05) of behavioural problems.
			Maternal depression				Maternal depression was significantly associated with onset (r = .17, p < .001) but not recurrence (r = .16, p > .05) of behavioural problems.
			Maternal hostility				Maternal hostility was significantly associated with the onset (r = .26, p < .001) and recurrence (r = .33, p < .01) of behavioural problems.
			Stressful life events				Stressful life events were not associated with the onset (r = .03, p > .05) or recurrence (r = −.03, p > .05) of behavioural problems.
			Number of children				Number of children was not associated with the onset (r = 0.4, p > .05) or recurrence (r = −.05, p > .05) of behavioural problems.
			Role conflict				Role conflict was significantly associated with onset (r = .15, p < .001) but not recurrence (r = .12, p > .05) of behavioural problems.
			Marital conflict				Marital conflict was significantly associated with onset (r = .10, p < .05) but not recurrence (r = .05, p > .05) of behavioural problems.
			Family income				Family income was significantly associated with onset (r = −.10, p < .05) but not recurrence (r = .14, p > .05) of behavioural problems.
Comeau and Boyle (2018)	Predictor: birth, 1–2 years, 3–4 years Outcome: 5–6 years	Social	Poverty trajectories	Income to poverty ratio was calculated by dividing family income by the poverty level for a family of its size	Externalising/internalising disorders (onset)	Behavioural problem Index	Children always in poverty showed more externalising and internalising problems than children intermittently in poverty (Δx2 = 193.67, df = 1, p ≤ .001; Δx2 = 50.54, df = 1, p ≤ .001). Children intermittently in poverty showed more externalising and internalising problems than children never in poverty (Δx2 = 51.13, df = 1, p ≤ .001; Δx2 = 60.91, df = 1, p ≤ .001).
D'Souza et al. (2019)	Predictor: 2 years Outcome: 4 years	Individual/Social	Prenatal stress	Perceived Stress Scale	Childhood behavioural difficulties (onset)	Strengths and difficulties questionnaire (SDQ)	Prenatal stress was not associated with the onset of behavioural difficulties (OR = 1.01, 95% CI = 0.98–1.03, p > .05).
			Prenatal depression	Edinburgh Prenatal Depression scale			Prenatal depression was not associated with the onset of behavioural difficulties (OR = 0.98, 95% CI = 0.65–1.47, p > .05).
			Postnatal depression and anxiety	Edinburgh Postnatal Depression scale/GAD-7			Postnatal anxiety was associated with onset of behavioural difficulties (OR = 1.68, 95% CI = 1.02–2.71, p < .05), while postnatal depression was not associated with onset (OR = 0.95, 95% CI = 0.57–1.55, p > .05).
			Maternal evaluation of child's behaviour	Evaluation subscale of “What being a parent of a baby is like” instrument.			Maternal evaluation was not associated with the onset of behavioural difficulties (OR = 0.99, 95% CI = 0.96–1.02, p > .05).
			Maternal formal/informal support	Family Support Scale			Maternal formal (OR = 1.01, 95% CI = 0.98–1.04, p > .05) and informal (OR = 0.99, 95% CI = 0.96–1.01, p > .05) support were not associated with the onset of behavioural difficulties.
			History of partner's verbal/physical conflict	Self-report item			History of verbal (OR = 1.08, 95% CI = 0.80–1.46, p > .05) and physical (OR = 1.20, 95% CI = 0.84–1.70), p > .05) conflict were not associated with the onset of behavioural difficulties.
Dunn et al. (2020)	Predictor: 0–24 months, 48–60 months Outcome: 120 months	Individual	Exposure to physical violence	Major life events inventory	Internalising symptoms (onset)	Child Behaviour Checklist (CBCL/6–18)	Exposure to physical violence in very early childhood was significantly associated with internalising (RR = 1.55, 95% CI = 1.25–1.92, p < .05) and externalising (RR = 2.18, 95% CI = 1.68–2.93, p < .05) symptoms. Exposure to physical violence in early childhood was significantly associated with internalising (RR = 1.65, 95% CI = 1.31–2.08, p < .05) and externalising (RR = 1.57, 95% CI = 1.22–2.01, p < .05) symptoms.
			Exposure to sexual violence				Exposure to sexual violence in very early childhood was significantly associated with externalising symptoms (RR = 2.59, 95% CI = 1.68–3.94, p < .05), but not with internalising symptoms (RR = 1.40, 95% CI = 0.91–2.16, p > .05). Exposure to sexual violence in early childhood was significantly associated with internalising symptoms (RR = 1.36, 95% CI = 1.04–1.79, p < .05), but not with externalising symptoms (R = 1.26, 95% CI = 0.85–3.94, p > .05).
Fitzsimons et al. (2017)	Predictor: 9 months, 3 years, 5 years Outcome: 5 years	Individual/Social	Poverty trajectories	N/R	Externalising/internalising disorders (onset)	Strengths and difficulties questionnaire (SDQ)	Persistence of poverty between 9 months and 5 years was associated with greater emotional, peer, conduct, and hyperactivity problems (all p < .01).
			Parental physical health	Malaise Inventory			Maternal longstanding physical health problem was associated with higher emotional and peer problems (both p < .10), while this was not the case with a father's longstanding physical health problem (both p = ns).
Hayatbakhsh et al. (2012)	Predictor: birth Outcome: 14 years	Individual	Breastfeeding	Self-report question at baseline not reported	Withdrawal/social/thought/somatic/attention/aggression/delinquency problems	Youth Self-Report (YSR) questionnaire	Children who were breastfed for at least 4 months reported fewer symptoms of anxiety/depression (p < .05), social problems (p < .001), attention problems (p < .001), aggression (p < .001), and delinquency (p < .001) at 14 years.
			Maternal age				Children whose mothers were younger than 20 years at pregnancy were more likely to report symptoms of aggressive (p < .001) and delinquent (p < .001) behaviour.
			Maternal education				Less educated mothers had children with more symptoms of social (p < .001), attention (p < .01), aggression (p < .001), and delinquency (p < .001) problems.
			Unplanned pregnancy				Children who were the outcome of an unplanned pregnancy were more likely to report problems with anxiety/depression (p < .05), withdrawal (p < .05), thought (p < .01), attention (p < .01), aggression (p < .001), and delinquency (p < .001).
			Maternal anxiety	Delusion symptoms states inventory (DSSI)			Anxious mothers were more likely to have a 14-year-old child with symptoms of anxiety/depression (p < .001), withdrawal problems (p < .001), social problems (p < .01), somatic problems (p < .001), thought problems (p < .001), attention problems (p < .001), aggression (p < .001), and delinquency (p < .01).
			Maternal depression				Depressed mothers were more likely to have a 14-year-old child with symptoms of anxiety/depression (p < .001), withdrawal problems (p < .01), social problems (p < .001), somatic problems (p < .001), thought problems (p < .01), attention problems (p < .05), aggression (p < .001), and delinquency (p < .01).
			Maternal smoking				Those who smoked cigarettes while pregnant were more likely to have a 14-year-old child with symptoms of anxiety/depression (p < .05), somatic problems (p < .01), thought problems (p < .05), attention problems (p < .001), aggression (p < .001), and delinquency (p < .001).
			Maternal alcohol use				Those who drank alcohol while pregnant were more likely to have a 14-year-old child with symptoms of anxiety/depression (p < .05), somatic problems (p < .01), thought problems (p < .001), attention problems (p < .001), aggression (p < .01), and delinquency (p < .001).
Hyland et al. (2016)	Predictor: birth Outcome: 10–21 years	Individual/Social/Urban	Advanced parental age	Older/younger than 40 years	Anxiety/mood disorders	ICD-10 diagnosis	Advanced paternal age was not a significant predictor of anxiety (OR = 0.97, 95% CI = 0.77–1.22, p = .77) or mood (OR = 0.94, 95% CI = 0.67–1.32, p = .71) disorders. Advanced maternal age was not a significant predictor of anxiety (OR = 0.81, 95% CI = 0.41–1.61, p = .55) or mood (OR = 1.25, 95% CI = 0.54–2.89, p = .60) disorders.
			Urbanicity	If the participant lived in any of the cities Aarhus, Odense, Aalborg, or Copenhagen			Urbanicity was a significant predictor of anxiety disorders (OR = 1.17, 95% CI = 1.06–1.29, p < .01), but not of mood disorders (OR = 0.89, 95% CI = 0.77–1.03, p = .12).
			Deprivation	Registers of Income compensation benefits, labour market research, and unemployment statistics			Parental unemployment was a significant predictor of anxiety (OR = 1.26, 95% CI = 1.13–1.41, p < .001) and mood (OR = 1.19, 95% CI = 1.01–1.41, p = .04) disorders.
			Parental self-harm	Hospital admissions			Parental self-harm was not a significant predictor of anxiety (OR = 1.19, 95% CI = 0.92–1.54, p = .18) or mood (OR = 0.92, 95% CI = 0.60–1.42, p = .71) disorders.
			Parental mood disorder	ICD-10 diagnosis			Paternal mood disorder was a significant predictor of anxiety (OR = 1.51, 95% CI = 1.12–2.03, p = .01) and mood (OR = 1.73, 95% CI = 1.14–2.63, p = .01) disorders. Maternal mood disorder was a significant predictor of anxiety (OR = 1.55, 95% CI = 1.21–1.97, p < .001) and mood (OR = 2.04, 95% CI = 1.46–2.86, p < .001) disorders.
			Parental anxiety disorder				Paternal anxiety disorder was a significant predictor of anxiety (OR = 1.84, 95% CI = 1.44–2.36, p < .001) and mood (OR = 1.65, 95% CI = 1.13–2.40, p = .01) disorders. Maternal anxiety disorder was a significant predictor of anxiety (OR = 2.38, 95% CI = 1.98–2.86, p < .001) and mood (OR = 1.97, 95% CI = 1.49–2.62, p < .001) disorders.
			Childhood adversity	Population-based register of social assistance for children in care			Child in care was a significant predictor of anxiety disorders (OR = 1.47, 95% CI = 1.17–1.85, p < .001), but not of mood disorders (OR = 1.07, 95% CI = 0.73–1.59, p = .72).
			Family dissolution	Danish Central Population Register			Family dissolution was a significant predictor of anxiety (OR = 1.64, 95% CI = 1.47–1.83, p < .001) and mood (OR = 1.53, 95% CI = 1.30–1.79, p < .001) disorders.
Karevold et al. (2009)	Predictor: 1–18 months Outcome: 12–13 years	Individual/Social	Family adversities	Questions referring to problems experienced during the last 12 months	Depression/Anxiety (onset)	Short Mood and Feeling Questionnaire - combined score parent and child report	Family adversities were associated with anxiety symptoms at t1 (r = .13, p < .01), t2 (r = .23, p < .01), and t3 (r = .13, p < .01) and with depression symptoms at t1 (r = .14, p < .01), t2 (r = .24, p < .01) and t3 (r = .19, p < .01).
			Maternal distress	Hopkins Symptom Check List			Maternal distress was associated with anxiety symptoms at t1 (r = .21, p < .01), t2 (r = .24, p < .01), and t3 (r = .18, p < .01) and with depression symptoms at t1 (r = .21, p < .01), t2 (r = .21, p < .01), and t3 (r = .19, p < .01).
			Social support	Index measuring social support received from close family, friends, and partners			Social support was associated with anxiety symptoms at t1 (r = −.13, p < .01), t2 (r = −.19, p < .01) and t3 (r = −.17, p < .01) and with depression symptoms at t1 (r = −.21, p < .01), t2 (r = −.23, p < .01), and t3 (r = −.25, p < .01).
Kiernan & Mensah, 2009	Predictor: 9–11 months Outcome: 36 months	Individual/Social	Poverty	Household income was 60 per cent below the median before housing costs	Behavioural adjustment (onset)	Strengths and difficulties questionnaire (SDQ)	Poverty at 9 months only (OR = 1.50, p < .05) was significantly associated with behavioural problems at 3 years.
			Maternal depression	Self-report item (e.g., “Since (the baby) was born, has there ever been a time lasting two weeks or more when you felt low or sad?“)			Maternal depression at 9 months only (OR = 1.50, p < .05), at 3 years only (OR = 2.67, p < .001), and at both 9 months and 3 years (OR = 3.75, p < .001) was significantly associated with behavioural problems, while maternal depression at neither 9 months or 3 years (OR = 1.00, p > .05) was not associated with behavioural problems.
			Family status	Survey N/R			Family status was not significantly associated with behavioural problems; married (OR = 1.00, p > .05), cohabiting (OR = 1.24, p > .05), lone parent (OR = 1.22, p > .05), stepfamily (OR = 1.31, p > .05).
King (2018)	Predictor: 3–5 years Outcome: 3–5 years	Social	Child food insecurity	8 items from the US Department of Agriculture Food Security Module	Internalising/externalising behaviours (onset)	Child Behaviour Checklist for ages 1.5–5 (CBCL)	Childhood food insecurity was significantly associated with internalising (p < .05) and externalising (p < .01) behaviours.
			Household food insecurity	18 items from the US Department of Agriculture Food Security Module			Household food insecurity was significantly associated with internalising (p < .01) and externalising (p < .05) behaviours.
Lai et al. (2019)	Predictor: 9 months, 3 years, 5 years, 7 years Outcome: 14 years	Social	Poverty trajectories	<60% of median household income where self-reported income	Socioemotional behavioural problems (onset)	Strengths and difficulties questionnaire (SDQ)	Exposure to poverty in early childhood was associated with socio-emotional behavioural problems (OR = 2.17, 95% CI = 1.68–2.80).
Leech et al. (2006)	Predictor: 0 months, 18 months, 36 months, 48 months, 72 months Outcome: 10 years	Individual/Social	Maternal marijuana use in first trimester	Average number of joints smoked per day	Depression and anxiety (onset)	Children's Depression Inventory (CDI) and Revised Children's Manifest Anxiety Scale (RCMAS)	Maternal marijuana use in the first trimester was significantly associated with child depression/anxiety at age 10 (RR = 1.63, CI = 1.06–2.49, p < .05).
			Household density	Paediatric Review and Observation of Children's Environmental Support and Stimulation (PROCESS), Home Screening Questionnaire, Home Observation for Measurement of the Environment (HOME)			Household density was significantly associated with child depression/anxiety at age 10 (RR = 1.19, CI = 1.05–1.35, p < .01).
			Church attendance	Maternal report			Church attendance was significantly associated with child depression/anxiety at age 10 (RR = 0.86, CI = 0.75–0.99, p <. 05).
			Race				Race was significantly associated with child depression/anxiety at age 10 (RR = 0.67, CI = 0.45–0.99, p < .05).
			No. of injuries				No. of injuries at 3 years was significantly associated with depression/anxiety at age 10 (RR = 1.41, CI = 1.00–1.99, p < .05).
			Mental development index	Mental Development Index of the Bayley Scales of Infant Development			Mental development index at 18 months was significantly associated with depression/anxiety at age 10 (RR = 0.97, CI = 0.96–0.98, p < .001).
			Composite IQ score	The Stanford-Binet Intelligence Scale			Composite IQ score at 3 years was significantly associated with depression/anxiety at age 10 (RR = 0.94, CI = 0.92–0.96, p < .001).
			Maternal depression	Centre for Epidemiological Studies Depression Scale (CES-D)			Maternal depression was significantly associated with child depression/anxiety at age 10 (RR = 1.02, CI = 1.00–1.05, p < .05).
Martikainen et al. (2018)	Predictor: 48 months Outcome: 300 months	Individual	Parental substance abuse	Tenth revision of ICD used to identify mental and behavioural disorders due to alcohol and substance use, alcohol-related diseases, toxic effects and poisoning by alcohol and other substances	Psychiatric morbidity (onset)	Psychotropic medication purchases or admission to inpatient hospital care with a psychiatric diagnosis (ICD-10) codes F10–69, F80–98	Exposure to parental substance abuse at 0–4 years increased the risk of subsequent psychiatric morbidity at the age of 15–25 (HR = 1.36, 95% CI = 1.25–1.47).
Najman et al. (2010)	Predictor: during pregnancy, 6 months, 5 years, 14 years Outcome: 14–21 years	Social	Family poverty	Total gross annual household income	Anxiety and depression (onset)	Youth Self-Report (YSR) questionnaire	Poverty during mother's pregnancy (p < .01), poverty at age 6 months (p < .05), and poverty at age 5 years (p < .01), were significantly associated with anxiety and depression at ages 14 and 21.
Shaw et al. (2016)	Predictor: 18 months, 24 months, 42 months, 60 months Outcome: 24 months, 42 months, 60 months, 72 months	Individual	Neighbourhood deprivation	US Bureau of Census data at the block group level	Conduct problems (onset)	Child Behaviour Checklist 2–3/Teacher Report Form (TRF)	Significant associations between neighbourhood deprivation at age 3.5 and conduct problems at age 5 (β = 0.09, p < .05), and between neighbourhood deprivation at age 5 and conduct problems at age 6 (β = 0.19, p < .05).
			Maternal depressive symptomology	Beck Depression Inventory (BDI), CES-D MD			Significant associations between mother's depressive symptoms at age 1.5 to conduct problems at 2 years (β = 0.22, p < .01), and between mother's depressive symptoms at age 2 to conduct problems a 3.5 years (β = 0.21, p < .01).

### 3.5 Individual-level risk factors

The studies included evaluated the effect of individual-level risk factors on the development of mood and anxiety disorders (n = 11), behavioural problems (n = 7), mental health difficulties (n = 1) and psychiatric morbidity symptoms (n = 1) in children and adolescents (n = 18). Transgenerational individual-level risk factors such as maternal psychopathology and exposure to familial violence were the most reported factors associated with the above-mentioned outcomes (n = 16).

### 3.6 Parental psychopathology

Five studies assessed whether parental psychopathology affected the risk of developing depression and anxiety in children, adolescents, and young adults. Maternal anxiety, distress and depression all significantly increased the chance of developing depression and anxiety in children, adolescents and young adults (age 6–21 years) (Hayatbakhsh et al., 2012; Hyland et al., 2016; Karevold et al., 2009; Leech et al., 2006).

Similarly, the risk of behavioural problems appeared to increase when parents experienced mental health problems themselves (Kiernan & Mensah, 2009, Cohen & Bromet, 1992; Christensen et al., 2017; Hayatbakhsh et al., 2012). Maternal anxiety was associated with the onset of behavioural problems in childhood (D'Souza et al., 2019) and adolescence (Hayatbakhsh et al., 2012). One additional study reported a positive association between the onset of conduct problems in childhood and maternal depression (Shaw et al., 2016). However, when maternal distress and psychopathology were assessed during pregnancy and birth, they showed a non-significant association with the onset of behavioural problems and mental health difficulties. Maternal distress during birth delivery was not associated with emotional or conduct problems in middle childhood (Bakoula et al., 2009), and prenatal maternal depression and stress were not associated with mental health problems in childhood (D'Souza et al., 2019). Overall, there appears to be an association between maternal psychopathology and the onset of behavioural problems, although this association is not present when the risk factor is assessed during pregnancy and at birth.

Substance use, in particular substance use by the mother, significantly increased the risk of developing depression, anxiety, somatic problems, thought problems, aggression and delinquency among children and adolescents (Hayatbakhsh et al., 2012; Leech et al., 2006). Parental substance use was also found to increase the risk of psychiatric morbidity in young adulthood (Martikainen et al., 2018). For instance, maternal marijuana use during the first trimester of pregnancy predicted depression and anxiety in middle childhood (Leech et al., 2006).

### 3.7 Family demographics

Mixed evidence emerged from the included studies when family demographics were explored as risk factor (n = 3). Maternal age was not associated with the onset or recurrence of behavioural problems (Cohen & Bromet, 1992), however, there was an association with delinquency and aggression when maternal age was below 20 years (Hayatbakhsh et al., 2012). When parental age was advanced, i.e. older than 40 years, a non-significant association was reported with anxiety and mood disorders (Hyland et al., 2016). Furthermore, maternal education was not associated with the onset or recurrence of behavioural problems (Cohen & Bromet, 1992), although it was associated with social and attention problems, delinquency and aggression (Hayatbakhsh et al., 2012). Finally, ethnicity was not associated with behavioural difficulties, however the likelihood of externalising disorders was greater among children who were not of minority race/ethnicity (Carter et al., 2010). Also, one study found that white ethnicity was positively associated with the onset of anxiety and depression in adolescence (Leech et al., 2006).

### 3.8 Violence exposure

The most explored factors among studies investigating family adversities (n = 5) were parental conflict and violence exposure. Both physical and sexual violence appeared to pose a risk to mental health, though in different ways. Physical violence exposure in very early (0–2 years) and early (4–5 years) childhood increased the odds of developing internalising and externalising symptoms in adolescence, while exposure to sexual violence in very early (0–2 years) and early (4–5 years) was associated with the development of externalising, but not internalising symptoms (Dunn et al., 2020). Also, exposure to sexual abuse in early childhood was significantly associated with internalising symptoms, but not with externalising symptoms (Dunn et al., 2020). Another study found that children who were exposed to physical violence were more likely to develop internalising symptoms (Carter et al., 2010).

### 3.9 Family environment

Family adversities such as partner relationship, use of alcohol and child rearing were associated with anxiety and depressive symptoms in middle childhood (Karevold et al., 2009). One study reported a positive association between parental role conflicts and the onset of behavioural problems in childhood (Cohen & Bromet, 1992). Another study found no evidence of behavioural problems in childhood when children were exposed to parental verbal conflict (D'Souza et al., 2019). This non-significant association could be explained by the fact that the outcome was measured at 4 years of age, at which point the child may be too young to understand and/or absorb parental verbal conflicts. Despite this non-significant association, these findings suggest that there may be a relationship between family adversities, violence exposure and the onset of symptoms of psychopathology in very young children, children and adolescents.

### 3.10 Parent-child interactions

Three studies explored the impact of parent-child interactions. There was mixed evidence for the impact that parent-child interactions might have on children's and adolescents' mental health. Conflictual or dependent parent-child interaction was not significantly associated with anxiety and depression in middle childhood (Barrios et al., 2018). This study also found that problematic sleep parent-child interaction, measured by the Parent Child Sleep Interaction Scale (PSIS), was not associated with the onset of anxiety and depression in middle childhood. Parenting style appeared to be associated with the same outcomes (i.e. onset of anxiety and depression). Low maternal warmth and high maternal hostility were associated with behavioural difficulties in adolescence (Christensen et al., 2017). Low family expressiveness was also associated with the onset of externalising disorders (Carter et al., 2010).

### 3.11 Other individual-level risk factors

Seven studies also reported findings for individual risk factors that could not be grouped into a broader category, as risk factors were few and conceptually heterogeneous. Leech et al. (2006) e.g. found a negative effect for IQ level and number of previous injuries on depression and anxiety in adolescence. Two studies investigated risk factors related to pregnancy and birth delivery. One study found that assisted delivery was associated with conduct problems in middle childhood, whereas caesarean section and child peripartum distress were not (Bakoula et al., 2009). Another study found that an unplanned pregnancy increased the odds of developing mental health problems in adolescence, and that breastfeeding for at least four months reduced anxiety, depression, social problems, attention problems, aggression and delinquency in adolescence (Hayatbakhsh et al., 2012). These effects were more pronounced for males than for females.

A longstanding maternal physical health problem was found to be positively associated with a child's emotional and peer problems, while this was not the case for a longstanding paternal physical health problem (Fitzsimons et al., 2017). Childhood adversities, defined as whether the child was placed into care or living in an institution, were shown to be associated with anxiety in middle adolescence and early adulthood (Hyland et al., 2016). Stressful life events experienced by the child were more likely to be associated with externalising problems in middle childhood (Carter et al., 2010). One study reported no association with the onset or recurrence of behavioural problems in childhood among mothers who had experienced stressful life events (Cohen & Bromet, 1992).

To summarise, the findings from studies exploring the impact of individual factors on the development of psychopathology (i.e., anxiety and depression, behavioural problems, and internalising and externalising symptoms) in childhood, adolescence and young adulthood are mixed. There is some evidence to suggest that parental psychopathology can increase the risk of developing depression and anxiety in children, adolescents, and young adults. Substance use, in particular substance use by the mother, was found to significantly increase the risk of developing depression, anxiety and psychiatric morbidity among children, adolescents, and young adults. Mixed evidence was found when family demographics such as age, education and ethnicity were explored as risk factors.

Of the studies included in this review that explored the impact of parental conflict and exposure to violence, all found that these factors had an impact on mental health, though in different ways. Findings from studies exploring family adversities such as partner relationship, use of alcohol and child rearing were mixed. Early life conflictual parent-child interactions do not appear to affect psychopathology; however, a hostile and low-warmth parenting style is associated with behavioural difficulties in adolescence. Finally, there is some evidence to suggest that factors related to pregnancy, birth delivery and childhood adversities are associated with the later development of behavioural and mental health problems in children and adolescents. Among studies investigating individual risk factors, only two were not rated as high quality as the exposure indicator was not systematically measured (Cohen & Bromet, 1992; Kiernan & Mensah, 2009).

### 3.12 Social risk factors

Social risk factors refer to the socioeconomic and household composition of the family, such as low socioeconomic status, social disadvantages, receiving public assistance and residential instability. The most investigated social risk factor was poverty, which was investigated by seven studies in this review. Two studies found that poverty in early childhood was associated with behavioural problems in adolescence (Christensen et al., 2017; Lai et al., 2019), with a further two studies finding an association with anxiety and depression in adolescence and early adulthood (Björkenstam et al., 2017; Najman et al., 2010). One study found that persistent poverty between 9 months and 5 years of age was associated with behavioural problems in childhood (Fitzsimons et al., 2017). Another study reported an association between children aged 0–4 years who grew up in poverty and internalising and externalising problems later in childhood (age 5–6) (Comeau & Boyle, 2018). A further study reported that poverty measured at 9 months was associated with the onset of behavioural problems at 3 years (Kiernan & Mensah, 2009).

The number of children in a household was not found to be associated with behavioural problems in childhood (Cohen & Bromet, 1992). Children growing up in single-parent households, along with those living in residential instability, appeared to have an increased incidence of depression and internalising problems in adolescence (Björkenstam et al., 2017). Another study reported that single-parent households were not associated with the onset of behavioural problems in childhood alongside other family statuses (e.g., divorced, single parents) (Kiernan & Mensah, 2009). Hyland et al. (2016) reported a significant association between family dissolution in early childhood and the onset of anxiety and mood disorders in middle childhood and adolescence. Household instability rather than family status may be the factor more strongly associated with mental health problems.

Social support in various forms appeared to have a positive and protective effect on mental health in two studies, whereas one other study did not find a significant association with the outcome measured. Receiving formal and informal social support outside the family was not found to reduce the likelihood of developing behavioural problems in childhood (D'Souza et al., 2019). One study specifically explored the effects of receiving household public assistance and found a significant association with internalising problems in adolescence (Björkenstam et al., 2017). The incidence ratios for the onset of internalising symptoms were lower when families received household public assistance solely during early childhood, in contrast to additionally receiving it during middle childhood and adolescence. Another study reported a significant association between child and household food insecurity and behavioural problems in childhood (King, 2018).

Of all the studies exploring social risk factors, only two studies assessing poverty were not rated as high quality as the exposure factors were not measured with a validated questionnaire (Najman et al., 2010; Kiernan & Mensah, 2009). The other studies investigating poverty were rated as high quality and the associations found were strong, as sample sizes were large, follow-ups covered childhood, middle childhood and adolescence and both exposure factors and outcomes were systematically measured.

### 3.13 Urban risk factors

Three studies included in this review explored urban risk factors (i.e. neighbourhood deprivation and urbanicity). Two of these studies specifically investigated the effects of neighbourhood deprivation in Australia and USA, defined as population data at the group level such as median family income, low educational attainment, high unemployment and percentage of undergraduate or postgraduate degrees (Christensen et al., 2017; Shaw et al., 2016). Economic and social disadvantages increased the likelihood of developing behavioural problems in adolescence (Christensen et al., 2017). Another study found that neighbourhood deprivation increased the likelihood of developing conduct problems in childhood (Shaw et al., 2016). Hyland et al. (2016) reported a positive association between urbanicity, measured as having lived in a major city in Denmark, and subsequent anxiety in adolescence and early adulthood. Overall, the studies included in this review suggest that growing up in an urban environment, particularly in a deprived geographical area, can increase the likelihood of developing symptoms of mental health problems in childhood, adolescence, and young adulthood.

## 4 Discussion

This review aimed to synthesize the literature on the possible effects of individual, social and urban factors, experienced during early childhood (age 0–5), on the development of later psychopathology (i.e. anxiety and depression, internalising and externalising symptoms and behavioural problems). We focused on children, adolescents, and young adults up to the age of 25. In our synthesis, twenty studies were identified that investigated the effects of exposure to individual, social and urban factors on later psychopathology. In particular poor socio-economic conditions (i.e. neighbourhood deprivation, parental psychopathology and exposure to violence) at a young age represent risk factors for later psychopathology and behavioural problems. Only one study explored interactions between factors, indicating scarcity of understanding how risk factors may affect each other.

In contrast to a growing body of literature on the relationship between urbanicity and mental disorders in adults (e.g. Van der Wal 2021), we found few studies that explored the association between early life exposure to urban risk factors and the later onset of psychopathology among children, adolescents and young adults. Moreover, only one study studied the interaction between two factors (Barrios et al., 2018). Only three studies investigated the effects of urban factors, specifically neighbourhood deprivation and urban dwelling, on children's, adolescents and young adults' mental health. They all reported a positive association between urban risk factors and the onset of symptoms of mental health problems across children's development. Neighbourhood deprivation and disadvantage in cities were found to be associated with the development of behavioural and conduct problems in childhood and adolescence (Christensen et al., 2017; Shaw et al., 2016), and an urban upbringing increased the likelihood of developing anxiety in adolescence and early adulthood (Hyland et al., 2016). Therefore, these findings suggest that early life exposure to a deprived urban environment, or urban upbringing, can affect children's and adolescents' mental health. However, more research is needed, given the scarcity of studies available.

While there is a lack of evidence on the relationship between the urban environment and mental health among children and adolescents, the literature focusing on adults suggests that a city's physical and social environments play an important role in the development of mental health problems (Krabbendam et al., 2021). Meta-analytical studies on adults have e.g. reported a positive association between air pollution, noise and depression (Braithwaite et al., 2019; Zeng et al., 2019). As our review suggests, the above-mentioned urban risk factors have been understudied in early childhood. Hence, it would be worth conducting future research into urban factors, especially on how they affect the development of psychopathology in adolescence and young adulthood. For instance, studies could consider assessing the effects of green spaces (Vanaken & Danckaerts, 2018) or violence reduction (Tuttle, 2021).

Our review also suggests that early life exposure to social risk factors, like social disadvantage, changes the risk for psychopathology. Poverty can act as a stressor for a child's caregiver, which might lead to stressor ‘feedback loops’, that can further amplify part of these problems (Van der Wal et al., 2021). For instance, low-paid parents must often work longer hours, which may not only heighten their own stress levels, but also reduce the available time to care for their children, that with a less predictable presence of parental care, often demands a higher level of responsibility with the child. In turn, these responsibilities might create an extra stressor for the child (Dashiff et al., 2009). Therefore, it is not surprising that six of the seven studies assessing poverty as a risk factor show a positive association with the development of behavioural problems, internalising and externalising disorders, depression and anxiety (Björkenstam et al., 2017; Christensen et al., 2017; Comeau & Boyle, 2018; Fitzsimons et al., 2017; Lai et al., 2019; Najman et al., 2010). The importance of a wealthy and stable social environment during early childhood is also shown by the positive associations found between residential instability, family dissolution and behavioural problems and anxiety (Björkenstam et al., 2017; Hyland et al., 2016). The mental health of children, adolescents and young adults may thus be threatened by stress related to poor socioeconomic conditions, which is usually more prevalent in deprived neighbourhoods (Castellani et al., 2015). Therefore, urban and social risk factors may intertwine as part of a larger complex system, in which social factors like unemployment and single-household living are affected by larger urban factors such as neighbourhood deprivation and vice versa.

Urban and social risk indicators are not the only risk factors associated with the later development of mental health problems. In the literature, the role of an adverse family environment in modulating the risk for mental disorders, like depression, is well-established (Otte et al., 2016). Therefore, beyond the risk factors already embedded in the urban and social environment, an adverse family environment can act at the individual level as an additional stressor for the mental health of very young children. Our current results suggest that maternal distress and other stress-related disorders, such as depression and anxiety, are associated with the onset of behavioural and conduct problems, depression and anxiety in adolescence and young adulthood (Kiernan & Mensah, 2009, Cohen & Bromet, 1992; Christensen et al., 2017; Hayatbakhsh et al., 2012; Leech et al., 2006; Karevold et al., 2009; Hyland et al., 2016; D'Souza et al., 2019). In addition to these transgenerational effects, exposure to violence and physical and sexual violence appear to be associated with the onset of behavioural problems and internalising symptoms (Dunn et al., 2020), whereas their impact on the onset of externalising symptoms is less straightforward (Carter et al., 2010).

In summary, early life exposure to an adverse family environment can enhance the risk to develop psychopathology in children, adolescents and young adults. In addition, interactive feedback loops and intergenerational effects of psychopathology might further increase the vulnerability of children to develop these adverse outcomes (Hayatbakhsh et al., 2012; Hyland et al., 2016; Karevold et al., 2009; Leech et al., 2006). Therefore, in addition to environmental risk factors that are present at an urban and social level, the likelihood of developing symptoms of mental health problems in childhood, adolescence and early adulthood is further heightened in individuals that are confronted by by early life familial and transgenerational risk factors.

### 4.1 Limitations

Our current review has several limitations. Screening the articles using ASReview software implies that we only screened 5% of articles from our searches. Although ASReview has been shown to perform well in finding articles that match the inclusion criteria, there is a possibility that some relevant articles were missed. In particular it is important to consider rank-order bias. In AI-aided pipelines, records are sequenced according to predicted relevance scores, potentially giving rise to a phenomenon known as rank-order bias (Gargon et al., 2019; Norman, 2020), where the arrangement of papers can influence decision-making. During screening prioritization, rank-order bias may emerge as screeners become influenced by the order of presented studies, potentially leading to the inclusion of non-relevant studies appearing first and the exclusion of pertinent ones listed later.

To mitigate this, we employed a three-phase screening process that goes beyond the standard PRISMA criteria for AI-aided screening with prioritization (Page et al., 2021). First, we used active learning using a shallow classifier with a pre-defined stopping rule to label the first set of papers (Brouwer et al., 2022). To avoid rank order bias, our second screening phase included a neural net learning model to identify papers that were difficult to identify due to concept ambiguity. The first and second phase utilised separate screeners, to avoid interpretation bias. After this, we conducted a third screening phase where a senior scholar double-checked the excluded references to correct any papers mis-labelled by the screeners of the first two rounds to avoid rank-order bias (Brouwer et al., 2022).

Secondly, the heterogeneity of statistical techniques used in the included studies and the limited number of studies for the urban and social levels meant that we could not carry out a meta-analysis. Conducting a meta-analysis would have been beneficial to explore the differential effects of different predictors. psychopathology.

Thirdly, we have been purposefully strict in our inclusion criteria. This includes only including outcomes that have been assessed using validated questionnaires, that were collected at specific timepoints. We note that our assessment timeframe (exposure between ages 0–5) and outcomes (between ages 5–25) may have limited the studies we were able to include.

Fourth, we did not find any study that investigated the effects of any risk indicator on well-being.

Finally, all studies were conducted in developed countries. Therefore, findings cannot be generalized to low and middle-income countries. Lastly, this review does not include studies investigating biological or genetic risk factors. As these risk factors also influence early life child's development, it has to be noted that including them would have returned an even more comprehensive picture of early childhood mental health risk factors.

### 4.2 Future research

Future studies should examine the association between early life exposure to urban risk factors and mental health in children, adolescents and young adults. Future studies should notably also focus on possible protective factors that can be present in an urban environment in early childhood, and their contribution to the development of psychopathology, as to our knowledge such studies are still absent. Literature regarding the association between urbanicity and mental health is already limited in the adult population, and it is almost absent when the target population involves very young children. This literature gap may be due to the difficulties in measuring exposure indicators in very young children. Moreover, there also is a lack of consistent measurement of specific urban factors, and the multitude of environmental effects on mental health has only recently been acknowledged (Van der Wal et al., 2021). Therefore, future studies should improve how, and at what timescale, urban indicators are captured for very young children. To do this, there is a need to explore potential data linkage options to cover existing data gaps between mental health and factors such as e.g. air pollution or urban heat. Finally, more research is needed on the association between the urban environment and mental health.

In order to fill this gap, there is firstly a need to move towards a standard conceptual definition of which urban indicators are considered critical, and an agreement on how these aspects of the urban environment can then be measured best. There have already been a few attempts to do this such as Brinkhof et al., (2022), who tested the validity of a single-item self-report measure for urbanicity, but more research is needed. Moreover, there is a need to better explore the intertwined relationship between urbanicity and mental health, characterised by dynamic processes and feedback interactions that unfold over time (Van der Wal et al., 2021). We recommend that future research addresses the dynamic interactions of urban indicators and mental health problems using conceptual and measurement techniques from the field of complexity science. Complexity science could aid to unravel the interaction of different indicators at different levels by modelling model non-linear and feedback effects (Van der Wal et al., 2021).

## 5 Conclusions

This review synthesises evidence of the impact of early childhood risk factors on anxiety and depression, behavioural problems, and internalising and externalising symptoms. While limiting the age range to between 0 and 5 years for the measurement of risk factors did restrict our final list of included studies, it also allowed us to identify which factors specifically predicted our outcomes of interest. Urban factors are understudied compared to individual level factors. Still, we do note that individual (parental psychopathology and exposure to family violence), social (poverty) and urban (urbanicity and deprivation) affect psychopathology in between ages 5–25. Future studies ought to explore interactions between individual, social and urban factors in order to further inform preventative approaches for common mental health conditions.

## Financial disclosure statement

This study was funded by the 10.13039/100007409Bernard van Leer Foundation, which had no involvement in the study design, collection, analysis, and interpretation of data, and in writing the manuscript.

## Ethical Statement for Solid State Ionics

Hereby, I/insert author name/consciously assure that for the manuscript/insert title/the following is fulfilled:
1) This material is the authors' own original work, which has not been previously published elsewhere.
2) The paper is not currently being considered for publication elsewhere.
3) The paper reflects the authors' own research and analysis in a truthful and complete manner.
4) The paper properly credits the meaningful contributions of co-authors and co-researchers.
5) The results are appropriately placed in the context of prior and existing research.
6) All sources used are properly disclosed (correct citation). Literally copying of text must be indicated as such by using quotation marks and giving proper reference.
7) All authors have been personally and actively involved in substantial work leading to the paper, and will take public responsibility for its content.

The violation of the Ethical Statement rules may result in severe consequences.

To verify originality, your article may be checked by the originality detection software iThenticate. See also http://www.elsevier.com/editors/plagdetect.

I agree with the above statements and declare that this submission follows the policies of Solid State Ionics as outlined in the Guide for Authors and in the Ethical Statement.

## Declaration of competing interest

None of the authors report a conflict of interest that relates to the contents of this manuscript.

## Figures and Tables

**Table:** Appendix 1 - Search string

#	Medline	PsycINFO	EMBASE	Scopus
1	mental health/OR mental health recovery/OR psychopathology/OR Happiness/OR mental distress/OR (happiness OR mental health OR mental symptoms OR mental illness OR distress OR wellbeing OR positiv* OR emotional regulation OR thriv* OR flourish*).ti,ab,kf.	psychopathology/OR well being/OR mental disorders/OR (mental disorder* OR psychiatric disorder* OR mental illness* OR mental health OR DSM* OR well?being).ti,ab,id.	mental health/or mental health recovery/or psychopathology/or Happiness/or mental distress/or (happiness or mental health or mental symptoms or mental illness or distress or wellbeing).ti,ab,kw.	TITLE-ABS (mental AND health OR psychopathology OR happiness OR distress OR mental AND symptoms OR mental AND illness OR wellbeing OR well-being OR dsm*) OR AUTHKEY (mental AND health OR psychopathology OR happiness OR distress OR mental AND symptoms OR mental AND illness OR wellbeing OR well-being OR dsm*)
2	precipitating factors/OR primary prevention/OR prognosis/OR protective factors/OR resilience, psychological/OR risk factors/OR risk/OR secondary prevention/OR tertiary prevention/OR determinant* OR etiolog* OR prosperitiy OR aetiolog* OR factors OR factor OR mediat* OR moderator* OR moderating OR moderated OR moderation OR precipitating OR predictor* OR prescriptive* OR predispos* OR prevent OR prevention OR preventative OR prognos* OR prospective OR protection OR protective OR protect OR reciprocal OR resilien* OR resistan* OR risk* OR susceptibility OR vulnerabilit* OR working mechanism*).ti,ab,kf.	etiology/OR prediction/OR prevention/OR prognosis/OR protective factors/OR relapse prevention/OR “resilience (psychological)"/OR risk factors/OR “substance use prevention"/OR “susceptibility (disorders)"/OR (determinant* OR etiolog* OR aetiolog* OR factors OR factor OR mediat* OR moderator* OR moderating OR moderated OR moderation OR precipitating OR predictor* OR prescriptive* OR predispos* OR prevent OR prevention OR preventative OR prognos* OR prospective OR protection OR protective OR protect OR reciprocal OR resilien* OR resistan* OR risk* OR susceptibility OR vulnerabilit* OR working mechanism*).ti,ab,id.	environmental factor/OR etiology/OR prediction/OR predictor variable/OR prevention/OR primary prevention/OR prognosis/OR protection/OR psychological resilience/OR recurrence risk/OR risk factor/OR secondary prevention/OR “social determinants of health"/OR tertiary prevention/OR (determinant* OR etiolog* OR aetiolog* OR factors OR factor OR mediat* OR moderator* OR moderating OR moderated OR moderation OR precipitating OR predictor* OR prescriptive* OR predispos* OR prevent OR prevention OR preventative OR prognos* OR prospective OR protection OR protective OR protect OR reciprocal OR resilien* OR resistan* OR risk* OR susceptibility OR vulnerabilit* OR working mechanism*).ti,ab,kw.	TITLE-ABS(determinant* OR etiolog* OR aetiolog* OR factors OR factor OR mediat* OR moderator* OR moderating OR moderated OR moderation OR precipitating OR predictor* OR prescriptive* OR predispos* OR prevent OR prevention OR preventative OR prognos* OR prospective OR protection OR protective OR protect OR reciprocal OR resilien* OR resistan* OR risk* OR susceptibility OR vulnerabilit* OR “working mechanism*") OR AUTHKEY (determinant* OR etiolog* OR aetiolog* OR factors OR factor OR mediat* OR moderator* OR moderating OR moderated OR moderation OR precipitating OR predictor* OR prescriptive* OR predispos* OR prevent OR prevention OR preventative OR prognos* OR prospective OR protection OR protective OR protect OR reciprocal OR resilien* OR resistan* OR risk* OR susceptibility OR vulnerabilit* OR “working mechanism*”)
3	late onset disorders/OR recurrence/OR remission, spontaneous/OR treatment outcome/OR (first-ever OR maintenance OR recover* OR onset OR persist* OR recur* OR relapse OR remission OR (return* ADJ3 symptom*) OR (symptom* ADJ3 reduc*) OR reoccurrence).ti,ab,kf.	“onset (disorders)"/OR persistence/OR “relapse (disorders)"/OR “remission (disorders)"/OR spontaneous remission/OR symptom remission/OR “recovery (disorders)"/OR symptom remission/OR treatment outcomes/OR (first-ever OR maintenance OR recover* OR onset OR persist* OR recur* OR relapse OR remission OR (return* ADJ3 symptom*) OR (symptom* ADJ3 reduc*) OR reoccurrence).ti,ab,id.	recurrence/OR remission, spontaneous/OR remission/OR mental health recovery/OR treatment outcome/OR (first-ever OR maintenance OR recover* OR onset OR persist* OR recur* OR relapse OR remission OR (return* ADJ3 symptom*) OR (symptom* ADJ3 reduc*) OR reoccurrence).ti,ab,kw.	TITLE-ABS(first-ever OR maintenance OR onset OR persist* OR recur* OR relapse OR remission OR (return* W/2 symptom* OR reoccurrence) OR (symptom* W/2 reduc*)) OR AUTHKEY(first-ever OR maintenance OR onset OR persist* OR recur* OR relapse OR remission OR (return* W/2 symptom*) OR (symptom* W/2 reduc*) OR reoccurrence)
4	animal/NOT human/	animal.po NOT human.po	(animal/OR animal experiment/OR animal model/OR nonhuman/) NOT human/	TITLE-ABS-KEY(animal* OR {nonhuman} OR mammal* OR “guinea pig*” OR monkey* OR {mouse} OR {mice} OR rabbit* OR {rat} OR rats OR rodent* OR {dog} OR {dogs} OR {cats} OR chicken*)
5	alcoholism/OR alcohol-related disorders/OR amphetamine-related disorders/OR behavior, addictive/OR cocaine-related disorders/OR heroin dependence/OR inhalant abuse/OR marijuana abuse/OR morphine dependence/OR narcotic-related disorders/OR opioid-related disorders/OR opium dependence/OR phencyclidine abuse/OR substance abuse, intravenous/OR substance abuse, oral/OR substance-related disorders/OR “tobacco use disorder”/OR agoraphobia/OR anxiety disorders/OR panic disorder/OR phobia, social/OR phobic disorders/OR depression, postpartum/OR depressive disorder, major/OR depressive disorder, treatment-resistant/OR depressive disorder/OR dysthymic disorder/OR mood disorders/OR seasonal affective disorder/OR (affective disorder* OR affective symptom* OR depress* OR dysthymi* OR mood OR anxiety OR acrophobi* OR agoraphobi* OR claustrophobi* OR phobi* OR panic disorder* OR addict* OR ((drug* OR substance OR 3-MMC OR 4-FA OR 4-FMP OR 4-MMC OR alcohol OR amphetamin* OR angel dust OR anxiolytic OR benzodiazepin* OR caffein* OR cannab* OR cigaret* OR cocain* OR ecstacy OR gamma hydroxybutyrate OR GHB OR hallucinogen* OR hashish OR heroin OR hypnotic OR inhalant* OR ketamin* OR LSD OR lysergic acid diethylamid* OR marijuana OR mdma OR methamphetamin* OR methylenedioxymethamphetamine OR nicotin* OR opiat* OR opiod* OR PCP OR phencyclidine OR sedative OR tobacco) ADJ2 (abus* OR depend* OR disorder* OR misus*)) OR alcoholis*).ti,ab,kf.	affective disorders/OR atypical depression/OR dysthymic disorder/OR late life depression/OR major depression/OR postpartum depression/OR recurrent depression/OR seasonal affective disorder/OR treatment resistant depression/OR acrophobia/OR agoraphobia/OR claustrophobia/OR generalized anxiety disorder/OR panic disorder/OR phobias/OR school phobia/OR social phobia/OR addiction/OR drug abuse/OR drug addiction/OR drug dependency/OR alcohol abuse/OR alcoholism/OR heroin addiction/OR inhalant abuse/OR polydrug abuse/OR (affective disorder* OR affective symptom* OR depress* OR dysthymi* OR mood OR anxiety OR acrophobi* OR agoraphobi* OR claustrophobi* OR phobi* OR panic disorder* OR addict* OR ((drug* OR substance OR 3-MMC OR 4-FA OR 4-FMP OR 4-MMC OR alcohol OR amphetamin* OR angel dust OR anxiolytic OR benzodiazepin* OR caffein* OR cannab* OR cigaret* OR cocain* OR ecstacy OR gamma hydroxybutyrate OR GHB OR hallucinogen* OR hashish OR heroin OR hypnotic OR inhalant* OR ketamin* OR LSD OR lysergic acid diethylamid* OR marijuana OR mdma OR methamphetamin* OR methylenedioxymethamphetamine OR nicotin* OR opiat* OR opiod* OR PCP OR phencyclidine OR sedative OR tobacco) ADJ2 (abus* OR depend* OR disorder* OR misus*)) OR alcoholis*).ti,ab,id.	adolescent depression/OR atypical depression/OR bipolar depression/OR chronic depression/OR depression/OR late life depression/OR long term depression/OR major affective disorder/OR major depression/OR mood disorder/OR perinatal depression/OR postnatal depression/OR recurrent brief depression/OR seasonal affective disorder/OR treatment resistant depression/OR acrophobia/OR agoraphobia/OR anxiety disorder/OR claustrophobia/OR generalized anxiety disorder/OR phobia/OR school phobia/OR social phobia/OR addiction/OR alcohol abuse/OR alcoholism/OR amphetamine abuse/OR amphetamine dependence/OR behavioral addiction/OR cannabis addiction/OR cocaine dependence/OR drug abuse/OR drug dependence/OR heroin dependence/OR inhalant abuse/OR intravenous drug abuse/OR morphine addiction/OR narcotic dependence/OR opiate addiction/OR phencyclidine abuse/OR multiple drug abuse/OR substance abuse/OR tobacco dependence/OR (affective disorder* OR affective symptom* OR depress* OR dysthymi* OR mood OR anxiety OR acrophobi* OR agoraphobi* OR claustrophobi* OR phobi* OR panic disorder* OR addict* OR ((drug* OR substance OR 3-MMC OR 4-FA OR 4-FMP OR 4-MMC OR alcohol OR amphetamin* OR angel dust OR anxiolytic OR benzodiazepin* OR caffein* OR cannab* OR cigaret* OR cocain* OR ecstacy OR gamma hydroxybutyrate OR GHB OR hallucinogen* OR hashish OR heroin OR hypnotic OR inhalant* OR ketamin* OR LSD OR lysergic acid diethylamid* OR marijuana OR mdma OR methamphetamin* OR methylenedioxymethamphetamine OR nicotin* OR opiat* OR opiod* OR PCP OR phencyclidine OR sedative OR tobacco) ADJ2 (abus* OR depend* OR disorder* OR misus*)) OR alcoholis*).ti,ab,kw.	TITLE-ABS(“affective disorder*” OR “affective symptom*” OR “depress*” OR “dysthymi*” OR {mood} OR {anxiety} OR acrophobi* OR agoraphobi* OR claustrophobi* OR “panic disorder*” OR “phobi*” OR addict* OR ((drug* OR substance OR “3-MMC” OR “4-FA” OR “4-FMP” OR “4-MMC” OR alcohol OR amphetamin* OR “angel dust” OR anxiolytic OR benzodiazepin* OR caffein* OR cannab* OR cigaret* OR cocain* OR ecstacy OR “gamma hydroxybutyrate” OR GHB OR hallucinogen* OR hashish OR heroin OR hypnotic OR inhalant* OR ketamin* OR LSD OR “lysergic acid diethylamid*” OR marijuana OR mdma OR methamphetamin* OR methylenedioxymethamphetamine OR nicotin* OR opiat* OR opiod* OR PCP OR phencyclidine OR sedative OR tobacco) W/1 (abus* OR depend* OR disorder* OR misus*)) OR alcoholis*) OR AUTHKEY(“affective disorder*” OR “affective symptom*” OR “depress*” OR “dysthymi*” OR {mood} OR {anxiety} OR acrophobi* OR agoraphobi* OR claustrophobi* OR “panic disorder*” OR “phobi*” OR addict* OR ((drug* OR substance OR “3-MMC” OR ”4-FA” OR “4-FMP” OR ”4-MMC” OR alcohol OR amphetamin* OR “angel dust” OR anxiolytic OR benzodiazepin* OR caffein* OR cannab* OR cigaret* OR cocain* OR ecstacy OR “gamma hydroxybutyrate” OR GHB OR hallucinogen* OR hashish OR heroin OR hypnotic OR inhalant* OR ketamin* OR LSD OR “lysergic acid diethylamid*” OR marijuana OR mdma OR methamphetamin* OR methylenedioxymethamphetamine OR nicotin* OR opiat* OR opiod* OR PCP OR phencyclidine OR sedative OR tobacco) W/1 (abus* OR depend* OR disorder* OR misus*)) OR alcoholis*)
6	(1 AND 2 AND 3 AND 4) NOT 5	(1 AND 2 AND 3) NOT 5	(1 AND 2 AND 3) NOT 5	(#1 AND #2 AND #3) AND NOT #5
7	6 NOT 4	6 NOT 4	6 NOT 4	#6 AND NOT #4
8	Limit 7 to english language	limit 7 to (peer reviewed journal and english language)	Limit 7 to (embase and english language)	DOCTYPE(ar) AND LANGUAGE(english)
9				INDEX(medline)
10				(#7 AND #8) AND NOT #9

## Data Availability

Data will be made available on request.
